# Autonomous Single-Molecule Manipulation Based on Reinforcement Learning

**DOI:** 10.1021/acs.jpca.2c08696

**Published:** 2023-02-07

**Authors:** Bernhard Ramsauer, Grant J. Simpson, Johannes J. Cartus, Andreas Jeindl, Victor García-López, James M. Tour, Leonhard Grill, Oliver T. Hofmann

**Affiliations:** † Institute of Solid State Physics, NAWI Graz, Graz University of Technology, Graz 8010, Austria; ‡ Department of Physical Chemistry, Institute of Chemistry, NAWI Graz, University Graz, Graz 8010, Austria; § Departments of Chemistry, Louisiana State University, Baton Rouge, Louisiana 70803, United States; ∥ Departments of Chemistry and Materials Science and NanoEngineering, and the Smalley-Curl Institute and NanoCarbon Center, Rice University, Houston, Texas 77005, United States

## Abstract

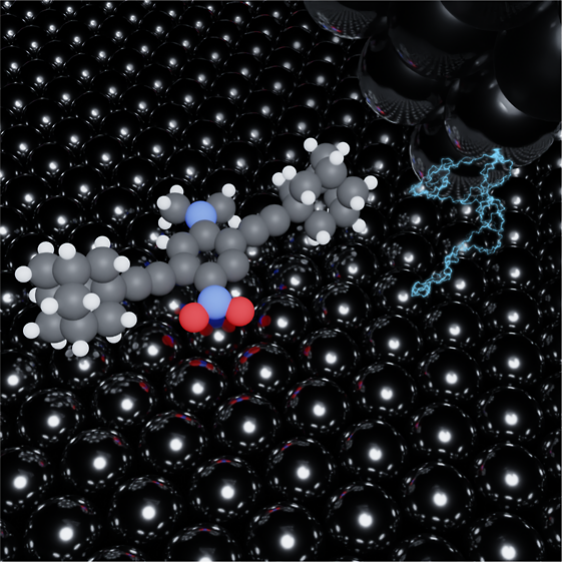

Building nanostructures one-by-one requires precise control of single molecules over many manipulation steps. The ideal scenario for machine learning algorithms is complex, repetitive, and time-consuming. Here, we show a reinforcement learning algorithm that learns how to control a single dipolar molecule in the electric field of a scanning tunneling microscope. Using about 2250 iterations to train, the algorithm learned to manipulate the molecule toward specific positions on the surface. Simultaneously, it generates physical insights into the movement as well as orientation of the molecule, based on the position where the electric field is applied relative to the molecule. This reveals that molecular movement is strongly inhibited in some directions, and the torque is not symmetric around the dipole moment.

## Introduction

Atomically precise control of the position and orientation of single molecules is key to improving the understanding of crystal growth and assembly processes, as well as the operation of molecular machines. From a more technological point of view, it also unlocks the possibility for nanofabrication of novel materials with enhanced properties that are inaccessible by means of conventional fabrication techniques. Presently, scanning probe methods (SPMs) are the preferred techniques capable of single atom or molecule manipulation, and many details about physical and chemical processes can be obtained only in this way at the single-molecule level.^1−6^

An established method to form nanostructures on surfaces is self-assembly due to lateral intermolecular interactions between molecules.^7,8^ Although those structures can be tailored by carefully selecting the functional groups of the molecular building blocks, arbitrary arrangements are not possible.^9,10^ Using the STM tip, custom-built atomically precise nanostructures on metal interfaces can be achieved by assembling individual atoms or molecules to create artificial structures,^11−13^ such as quantum corrals^14−16^ or 2D-materials.^17,18^ Even nanoelectronic computational devices, like logic gates,^19−21^ can be constructed from molecules by controlling their position and orientation. However, building even relatively small nanostructures requires hundreds or even thousands of manipulation steps. Performing all these manually is challenging on a routine basis.

Ideally, such jobs should be automized, and the easy interfacing of modern machines with programming languages allows algorithms to perform complex tasks. However, algorithms that are deterministic or based on fixed decision processes require a priori knowledge about the outcome in order to perform a meaningful action. Such information is typically unavailable when dealing with individual molecules on surfaces but can be obtained with a dynamic machine learning algorithm capable of learning from each performed manipulation. A pre-requisite to allow learning of these interactions, however, is that the interaction remains unaltered during the manipulation processes. For SPM techniques, this is not always true. At close tip-surface distances, molecules can be pushed over the surface but this can lead to strong interactions and subsequent tip changes.^4,8,22^ Note that machine learning approaches exist for in situ tip conditioning^23−25^ and tip apex classification using convolutional neural networks.^26^ However, these are not required for our approach, because the long-range interaction of the STM-tip-induced electric field and the dipole moment of the molecule hardly ever change the shape of the tip. This enhances the ability to acquire knowledge and allows to autonomously learn the individual manipulation parameters by systematically exploring the possible actions, for example, by exploring how much and in which direction a molecule would move if an electric field is applied at specific positions with respect to the molecule. Alongside the large number of possibilities to place the tip, the situation is complicated by the fact that interactions in the quantum world are often stochastic, that is, the same action may not always lead to the same outcome. In other words, obtaining this information and building an algorithm that allows the assembly of building blocks on the surface into arbitrary structures require a lot of repetitive and time-consuming tasks, which must be performed anew for every new type of building block (and for every surface on which the molecule should be manipulated).

Machine learning algorithms provide a promising solution to this kind of problem, and they have already demonstrated their capabilities for a variety of tasks: they can solve highly complex computer games at super-human performance,^27−30^ even when the rules of the game are a priori unknown. In more science-related context, machine learning has been integrated to simulate environments,^31−33^ autonomous data acquisition in SPM experiments,^34,35^ and the detection and movement of nanowires using an atomic force microscope.^36^ The notable capabilities of STMs to autonomously assemble atoms into atomically perfect nanostructures have been demonstrated.^11^ In their work, single Co adatoms are manipulated by creating a temporary bond between the adatom and the tip and then moving the adatom along a predetermined trajectory determined by a path planning algorithm. In addition to these experiments, reinforcement learning approaches were realized in scanning tunneling microscopy that are complementary to our manipulation approach, for example, to disassemble layers of organic molecules by bringing the STM tip in the vicinity of the molecule and extract it^37^ or manipulating single silver atoms precisely toward specific positions on a Ag(111) surface.^38^

In this work, we demonstrate a reinforcement learning approach that learns to maneuver individual molecules to a certain position on the surface utilizing a tip-induced electric field. After about half a day of training, the algorithm can manipulate molecules efficiently toward arbitrary positions on the surface. Additionally, it provides insight into the behavior of the molecule based on the relative position of the STM tip.

## Methods

The molecules [2,5-di(ethynyladamantanyl)-4-(dimethylamino)nitrobenzene (DDNB)] were deposited from a Knudsen cell at a temperature of 389 K onto a clean Ag(111) surface for 7 min under UHV conditions (1 × 10^–10^ mbar). Our experiments were performed using a low-temperature STM (CreaTec) operated at 5 K, and the STM tip is an electrochemically etched tungsten wire, likely covered with silver atoms after many tip indentations for routine cleaning. Once cooled, a molecule is extracted from an island by STM manipulation and moved to an area that is relatively clear of other molecules, adsorbates (like CO), or surface defects like step edges (see Supporting Information Figure S1). The STM is controlled *via* the component object model interface to communicate between the machine learning algorithm (i.e., our machine learning algorithm written in Python) and the key, value-based interface command structure of the STM. A more detailed description of the interfacing is given in the Supporting Information. During the STM manipulation, the bias voltage and tunneling current were fixed to 1.7 V and 11 pA, respectively, and the height of the tip 1.0 Å above the surface, while the lateral tip position was changed.

## Results and Discussion

**Figure 1:**
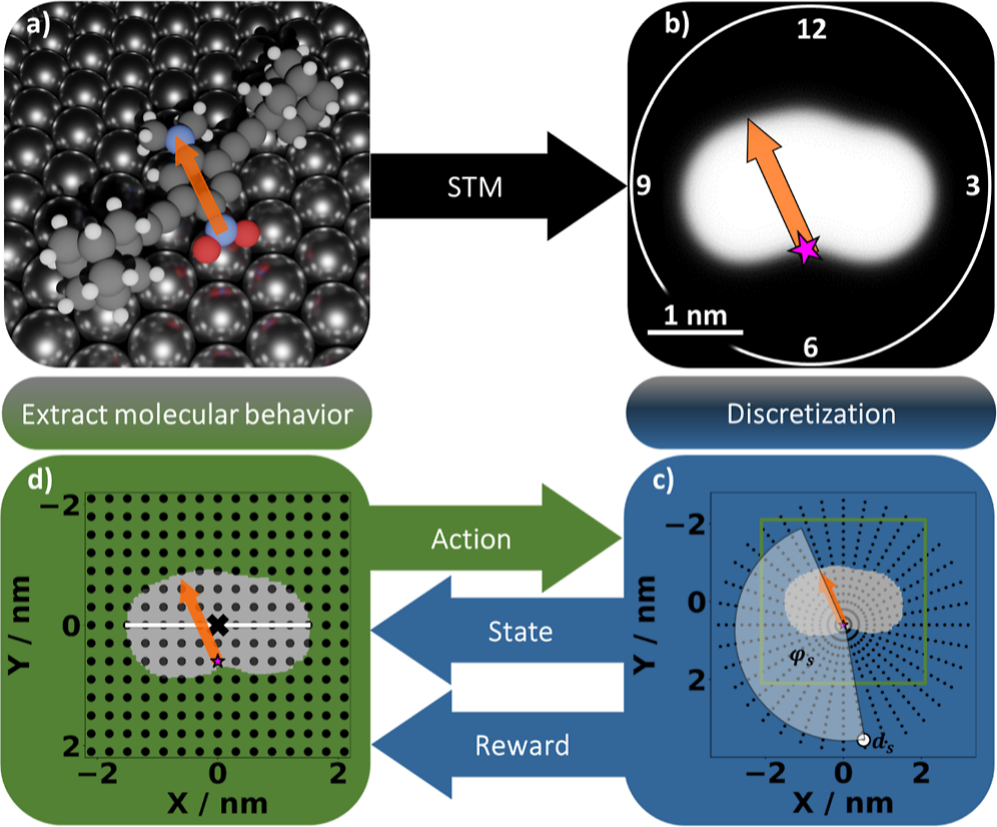
Sequential autonomous learning procedure is done by analyzing the STM image and extracting the molecule’s position and orientation, which, after a discretization step, represents the state of the system given to the reinforcement learning algorithm. (a) 3D structure of the molecule adsorbed on the Ag(111) surface: the colored NO_2_ group (pivot point) and the N(CH_3_)_2_ group on the para-position of the phenyl ring form the dipole of the molecule. This phenyl ring is connected to two adamantane wheels that lift the chassis off the surface. (b) STM image of the molecule is shown (tunneling parameters: 1 V, 11 pA). (c) Complete state-space as characterized *via* the discretization into 15 distances d and 360 angles φ (for visualization purposes, only 36 discrete angles are shown per distance *d*_s_). A single goal (white circle) is given by the distance *d*_s_ and angle φ_s_ from the molecule’s pivot point (indicated as “*”) to the goal position. The green square visualizes the size of the action space. (d) Action space is formed by a grid of 15 × 15 tip positions around the center of the molecule (indicated as “*x*”) such that the *x*-axis is aligned with the axis of the molecule (white line). The basic principle of reinforcement learning is given by combining (c,d).

In this work, we exemplarily use the manipulation of single DDNB (see Figure 1a) on Ag(111) to demonstrate an approach that can reliably control molecular movement using a tip-induced electric field. We emphasize, however, that our algorithm could be applied to any polar molecule, without prior knowledge of the atomistic structure of the molecule. The only input we provide is the “shape” of the molecule, that is, how it appears in an STM image, to facilitate its recognition by the algorithm. The choice of the molecule is motivated by earlier experiments, which also used this molecule for the same purpose.

These earlier studies showed that DNBB bears an intrinsic dipole moment arising from the electron-accepting nitro- and electron-donating dimethylamine group attached to the central phenyl ring, while being slightly elevated (and, thus, electronically decoupled) from the surface through the adamantyl side groups. Its dipole allows for molecular manipulations *via* the electric field localized around the STM tip and has been studied before.^39,40^ However, in principle, other effects such as inelastic excitation may also contribute to the movement of the molecule. This is of no further consequence to the algorithm, which makes no a priori assumption on the interaction or its mechanism. The forces at work can only be determined in hindsight after analysis of the data, as we show in the final section of this contribution.

The target of our approach is to autonomously move the molecule to a pre-determined position on the surface (hereafter called “goal”) by controlling the STM *via* a so-called intelligent agent,^41^ that is, something that perceives its environment (real world) and autonomously acts on it (through the STM) to achieve its goal. Earlier experiments in combinations with DFT calculations also showed that the NO_2_ group is in direct contact with the surface and acts as pivot point, that is the molecule rotates around this point at low voltages (1.3 V).^39^ At higher voltages (here, we use 1.7 V), also translation of the molecule, in addition to rotation, occurs—both strongly depending on the precise experimental settings, in particular the position of the STM tip during manipulation.^39^

For each pulse, the tip moves laterally to the desired location using the feedback set-point parameters *U* = 1 V, *I* = 11 pA. Subsequently, the feedback is deactivated, the tip is moved 1 Å closer to the surface, and the bias voltage is set at 1.7 V. After some time, a current exceeding 1 nA is observed and indicates that the molecule has moved which leads to automatic deactivation of the voltage pulse. The parameters are chosen because previous experiments show that these pulses frequently (though not always) induce a translation in the molecule, without destroying it.^39,40^

We note in passing that it would be interesting (and possible) to also let the algorithm autonomously determine the pulse settings. However, this would significantly increase the number of possible actions and requires a robust routine to determine whether the molecule is still intact or even present on the surface, which is beyond the scope of the present work.

Before each manipulation, the molecule is imaged (using the parameters described in Figure 1b) to analyze its topography and determine the position and orientation of the molecule (see Supporting Information: Figure S2). In order to manipulate the molecule, the agent varies the position of the tip relative to the molecule before the pulse is applied.

In reinforcement learning, the set of all possible distances and orientations where the goal can be located are known as the “state space” (black dots in Figure 1c). In our case, a single “state” is determined by the distance *d*_S_ between the goal and the pivot point of the molecule (indicated as pink “*” in Figure 1b), as well as the angle φ_S_ between the dipole moment and the vector from the pivot point to the goal (white dot in Figure 1c). To move the molecule across the surface, we use “actions”. Actions are manipulations of the molecule *via* voltage pulses at specific tip positions. The set of all possible actions (referred to as “action space”) is defined as tip positions arranged on a regular grid (Figure 1d) around the center of the molecule (black *x*), such that the *x*-axis is aligned with the connecting line between the two adamantane wheels (mass axis) of the molecule (white line). The combination of (c,d) in Figure 1 shows the basic principle of reinforcement learning.

To find the best action (i.e., the action that moves the molecule closest to the goal) for a given position and orientation of the molecule relative to the goal, it is necessary to communicate the quality of each action (determined by a reward function) to the agent. In general, we want to reward the agent for movements toward the goal that are larger than a minimum distance and explicitly penalize it for movements which are small or lead away from the goal. For this, we define the reward as a piecewise linear function (eq 1) such that the reward depends on the distance the molecule moves toward the goal, Δ*d* = *d*_*t*+1_ – *d*_*t*_. For technical reasons, it is useful to restrict the reward to values between −1 and +1. Thus, we normalize the distance by dividing it by a constant number *a*_max_. Empirically, we find *a*_max_ = 2.1 nm to be a good value here, motivated by the maximum distance the STM tip can be placed away from the molecule and still being able to induce molecular motion. Larger travel distances than *a*_max_ hardly ever occur, and if they do, the associated reward is capped at a value of 1. Conversely, a reward of −1 (i.e., a penalty) is assigned not only if the molecule ends up further away from the goal than it started but also if the molecule does not move at all (both cases represent an unsuccessful action). The reward is, apart from the state, the only information the agent receives. Therefore, both the behavior of the agent and the objective we want to achieve are encoded in this reward function. Mathematically, the function is given as
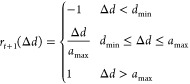
1

These are the necessary ingredients to formulate a reinforcement learning algorithm as a set of finite Markov Decision Processes (MDPs).^42^ A finite MDP is defined as a 4-tuple (*S*, *A*, *P*_*a*_, and *R*_*a*_), with *S* and *A* being the state space and action space, respectively, and the reward for a corresponding action *a* is given by *R*_*a*_. At any iteration, *t*, the system is in a state, *s*_*t*_. The agent performing an action, *a*_*t*_, brings the system with a probability, *P*_*a*_ = *p(s*_*t*+1_|*s*_*t*_, *a*_*t*_), to the next state, *s*_*t*+1_. After action *a*_*t*_ is performed, it is attributed a reward signal r_*t*+1_ (see eq 1) to determine the quality of the performed action. As a result, the agent learns a mapping between states and actions, which are formally described as state–action pairs. These state–action pairs are updated after every performed action, formally known as temporal difference learning TD(0), where the number describes how many actions, after the initial action, have to be performed until the reward is applied. The values of this map are stored in a lookup table.

Because the entries (i.e., the *Q*-values) of this *Q*-table are initially unknown, we train the agent using a strategy known as *Q*-learning.^43^ We note that RL methods are often realized as a neural network when the system requires continuous state–action space, which also makes them too large to be handled *via* table-based methods. Since this is not the case for our system, we found the *Q*-learning approach to be an ideal solution. Here, the *Q*-value for a state–action pair is determined by the Bellman optimality equation
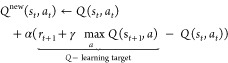
2

This equation contains two free hyperparameters: the learning rate α and the discount factor γ. The learning rate determines the weight between newly acquired information and already learned *Q*-values and essentially determines how important the present knowledge is relative to the newly obtained reward. Smaller values of α therefore enhance convergence but lead to a smaller step size toward a steady *Q*-value, while larger values allow for larger steps but may lead to increased instability.^44^ The discount factor γ determines how impactful already established *Q*-values are compared to newly obtained ones, which may be obtained coincidentally in stochastic experiments.^41^ During the training of the algorithm, we choose a learning rate of α = 0.3 and a discount factor of γ = 0.5. The choice of these hyperparameters is given by the assumption about the environment (i.e., how the molecule is interacting with the electric field). The small γ value leads to a very short-sighted agent because we thought the high voltage to induce molecular motion would inevitably cause uncontrollable rotations. In hindsight, this turned out not to be correct (see below), which means that better hyperparameters could be chosen in future experiments. The high learning rate, however, can lead to overfitting but is most likely suppressed by the high exploration rate of 70%.

A major challenge of our approach is the size of the *Q*-table. We allow for a total of 5400 possible states, given by 360 possible orientations toward the goal (i.e., 360 possible angles in 1° increments, see Figure 1c) and 15 possible distances (i.e., pivot-point to goals). Furthermore, we consider 225 possible actions, that is, tip positions relative to the molecule, on a regular grid of 4.2 nm by 4.2 nm, with a lattice spacing of 0.3 nm (see Figure 1d). Clearly, it is impractical to visit all possible state–action pairs, since this would require more than 1.2 million actions (position the tip, apply the voltage pulse, and image the position of the molecule) to visit every *Q*-table entry at least once.

Instead, we implement “virtual” states to speed up learning. In the experiment, before every action, the system is in one particular state that is defined by the distance and the orientation of the goal relative to the molecule. Based on this state, a certain action is taken, during which the molecule moves (or not), and after which the molecule is in a new state. To speed up learning, we employ a trick: we can treat the analysis as if the goal was in a different position (i.e., initially the system was in a different virtual state). Then, the action was taken (this is fixed because this is the experiment that was performed). Afterward, we can again determine the virtual state the system is in if the goal was at that position and thus evaluate the outcome (reward) of this action. Thus, instead of only having one goal (i.e., the real goal), we can define virtual goals such that all possible states (i.e., the whole state space) are presented to the agent simultaneously. Subsequently, we calculate the reward for all the different, virtual states which allows us to update 5400 entries of the *Q*-table accordingly.

**Figure 2:**
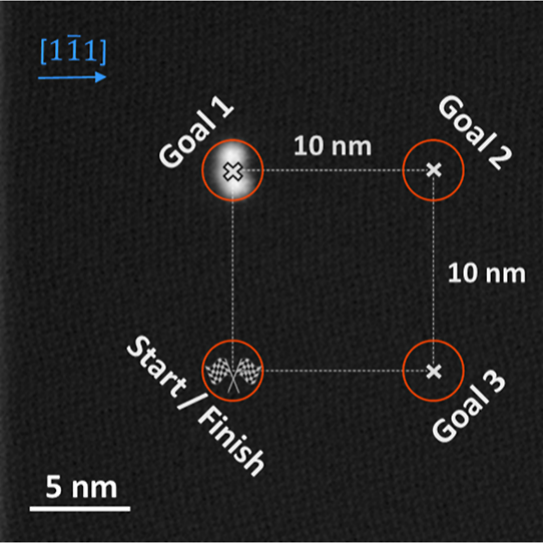
Learning and validation trajectory consisting of a square with 10 nm side length superimposed on an STM image (1.00 V, 0.11 pA) of a single molecule on Ag(111). The goal areas are circles with a radius of 1.5 nm at the corners of the square. The agent must reach the goal areas sequentially as indicated by the goal numbering. The STM image shows the molecule (at goal 1) for size comparison.

For the actual training, we maneuver the molecule along a square-shaped trajectory (shown in Figure 2), where the surface area is perfectly flat and without defects. The trajectory is defined by four goal regions on the surface (orange circles in Figure 2), which the molecule should reach sequentially. The points are shaped in a square, such that the molecule traverses different crystallographic directions of the hexagonal Ag(111) surface. We emphasize that apart from setting up four goal positions, the whole experiment (i.e., the training as well as validation runs) occurs in a fully automated manner. The software automatically analyzes the topography (see Figure S2 in the Supporting Information) to determine the state of the molecule on the surface (i.e., its distance and orientation relative to the goal), and also the action, that is, the placement of the tip for the voltage pulse, is done without human interference.

To train our algorithm efficiently and prevent the molecule from moving too far away from the square trajectory, it is necessary to find a trade-off between efficiently moving toward the goal and exploring actions that have not been tested yet. To do so, we defined the rate at which the agent explores new actions, that is, the exploration rate ε. At each step, our algorithm chooses with a 30% probability the action which is presently known to move the molecule most efficiently to the target, that is, the largest entry of *Q* for the present state. With ε = 70% chance, it explores the action space by using an action that gives the most information about the action space.

The action which yields the most information is determined *via* modeling the action space with Gaussian process regression (GPR) and selecting the action of highest GPR uncertainty (see Supporting Information). Note that even when the optimal action for the present state is chosen, at the same time sub-optimal actions for other (hypothetical) states are consistently explored because we analyze the data after each action with all possible “hypothetical” goal positions. This allows us to get a comprehensive and statistically well-sampled overview over the possible actions.

**Figure 3:**
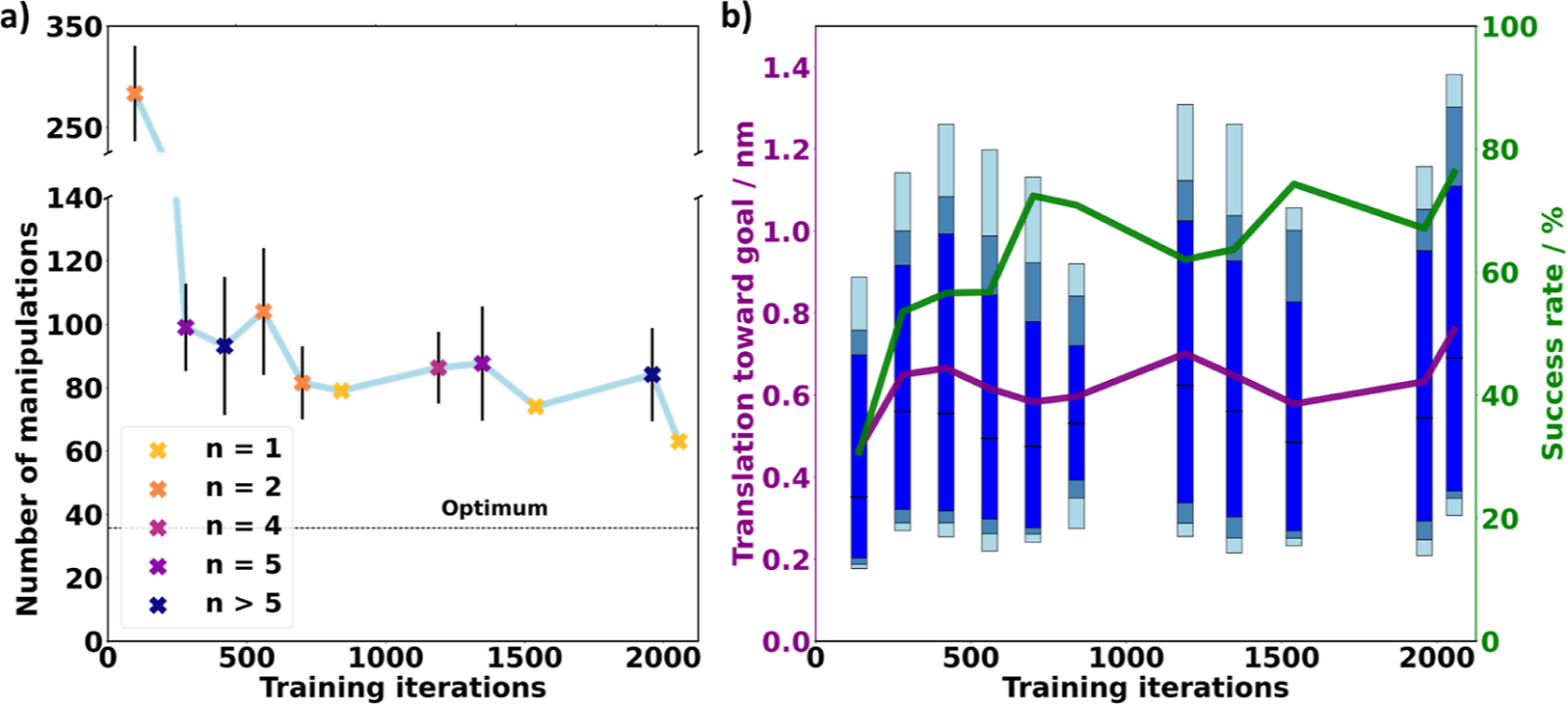
Learning progression. (a) Average number of manipulations necessary to complete the square trajectory used while training. The number of times *n* a validation run is performed at a given learning progression is indicated by the point coloring. The dotted line represents how fast the track could be completed if every manipulation induces a 1.4 nm movement. (b) As learning progresses, the mean distance (purple) the molecule moves toward the goal and the success rate (green) that the individual manipulations bring the molecule closer to the goal are shown. The box plots show the spread of the distance the molecule moves toward the goal for 60, 70, and 80% of the successful actions color coded from dark to light blue, respectively.

After a certain number of iterations, we interrupt the training of the algorithm to record its present performance with a set of validation runs. During these validation runs, we visit the same four goals multiple times but set the learning rate α to zero and only use the optimal *Q*-values for the present (real) state. Figure 3 shows the results of these validation runs. In Figure 3a, the number of actions required to finish a full square is given as a function of the training time, while the color of the data points indicates the number of times a validation run is performed at a given learning progression. We see that training varies between different runs of the same set, attesting to the stochastic nature of the procedure and the fact that the molecule cannot be moved equally successful in all directions. This is clearly shown in the first two validation runs, which are done only after 140 training iterations, which require 150 and 350 actions, respectively, to complete a full square. Already at the second validation set (after 280 learned iterations), the algorithm consistently completes a square in less than 125 actions (indicated by also taking the error bars into account).

This stochastic nature of the interactions is shown in Figure 3b, where the average distance the molecule moves toward the goal exhibits a large spread within each validation run. This spread is shown *via* box plots for 60, 70, and 80% of the successful actions. The purple line represents the average movement distance toward the goal. We note in passing that part of this spread also comes from the fact that translation and rotation around the pivot point are superimposed, as discussed in more detail in Figure S3 of the Supporting Information. Nonetheless, naively, one would expect that the average movement distance consistently increases as the learning progresses. Interestingly, this is the case between the first and the second validation set, but after that, the average movement distance is roughly alternating until the agent learned for about 2000 iterations. We find that the success rate, that is, the percentage of pulses that lead to a movement of the molecule toward the goal (shown as green line in Figure 3b) shows a similar trend to the movement distance, but in an anti-correlated manner.

This is owed to the way our algorithm is set up. In its entirety, the agent learns actions that complete the overall trajectory in less iterations while also being more reliable. The interesting behavior is revealed by taking a closer look at the individual learning progressions. After 280 learned iterations are completed, it starts to trade between actions that are fast (i.e., move the molecule over large distances) yet unreliable versus actions that move the molecule by a smaller distance, but do so more consistently. In the second training cycle, it trades off large movements that are less likely to be successful against smaller movements that are more reliable. This anticorrelation between distance and reliability continues until the second last training cycle. In the last training cycle, it finds actions that move the molecule reliably over large distances. This is a direct consequence of the designed reward function. Moderate movements toward the goal generate only relatively small rewards (*r* ≪ 1), while not moving at all is harshly penalized (*r* = −1). The agent heavily penalizes actions for not moving the molecule toward the goal, consequently decreasing its corresponding *Q*-value entry such that it is not the highest *Q*-value (i.e., best action in this state) anymore. This disincentivizes the algorithm to rely on actions whose outcome is strongly stochastic and pushes it toward well-defined, reproducible actions. Although this is hard to converge, in the end, it leads to faster and more reliable movements compared to all previous validation runs.

To judge the quality of the training, it is important to consider how fast the track could be completed under ideal circumstances, which is only possible in hindsight by analyzing the data. The circumference of the square amounts to 40 nm. As Figure 3b shows, the largest movement of the molecule that can be induced with our settings amounts to approximately 1.4 nm, that is, at least 29 manipulations would be required for the whole track. However, as shown below, this requires an optimal orientation of the molecule toward the goal, which we presently do not control. More importantly, as also shown in Figure 3b, even in the best circumstances, only about 80% of the pulses induce translation at all, due to the stochastic (quantum-mechanical) nature of the interaction. Thus, taken the best possible circumstance into consideration, a full square can realistically be completed with about 36 attempted manipulations (see dashed line in Figure 3a). Our fully trained algorithm approaches about 63 manipulations (i.e., 1.75× the optimum value) reasonably fast. This shows that our chosen approach (*Q*-learning) is, in the present case, sufficiently good to deal with this system, even if more sophisticated approaches, such as policy gradient methods, could improve the learning behavior even more.

**Figure 4:**
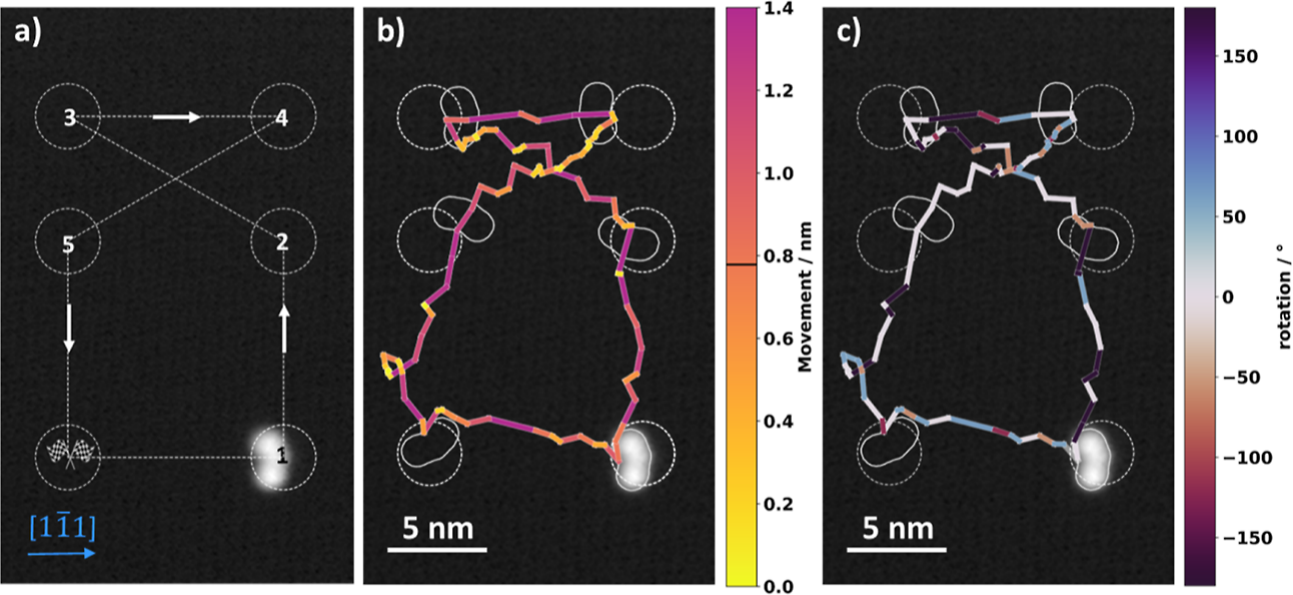
(a) Trajectory is defined by six goal positions (circles) forming a fish-shaped trajectory. (b) Final performance of the agent along a fish-shaped trajectory. The molecule is maneuvered sequentially through the goal regions (1, 2, 3, ...) indicated by gray circles. The distance traveled within each manipulation is indicated *via* its color. The average distance the molecule moves per manipulation is about 0.77 nm, but many successful movements are much larger (purple lines). The agent moves the molecule with an average success rate of 82% across the trajectory. (c) Rotation of the molecule for each manipulation is indicated *via* its color, whereas positive and negative angles correspond to counterclockwise and counterclockwise rotation, respectively. STM image: (1.00 V, 11 pA).

With the algorithm successfully trained on a square arrangement of points, the question arises whether it is now limited to specific kinds of translations on the surface. To show that we can efficiently manipulate the molecules to arbitrary points on the surface, we trained the algorithm for another 200 iterations (along the squared training trajectory) and then designed a track that roughly resembles the shape of a fish (see Figure 4a). This shape was chosen because the shortest path between some of the points (2–3 and 4–5) is along different crystallographic directions than the paths used in the training of the algorithm. Figure 4b shows how the algorithm moves the molecule across the surface, color-coding the distance it traveled with each manipulation. Encouragingly, we find that almost all manipulations are successfully steered toward the goal, and the agent was able to manipulate the molecule with an average success rate of 82% toward the goal which was even higher than that in all the previous validation runs reaching an average movement toward the goal of 0.77 nm per manipulation. The rotational behavior of the molecule after each manipulation is color-coded (see Figure 4c) and shows contrary to our expectation (that the molecule rotates randomly on top of the translation) that in 50% of the performed manipulations, no rotation is induced. We note that the target accuracy we defined, which is a 1.5 nm radius around the goal, was chosen very pragmatic to half the molecule’s width. In hindsight, we find that because we can systematically induce small or large translations, a smaller target radius could easily be chosen in future experiments.

The number of training iterations (2256), which by itself can be easily obtained within a short amount of time (about half a day), are sufficient to allow the code to move the molecule reproducibly to arbitrary points on the surface.

**Figure 5:**
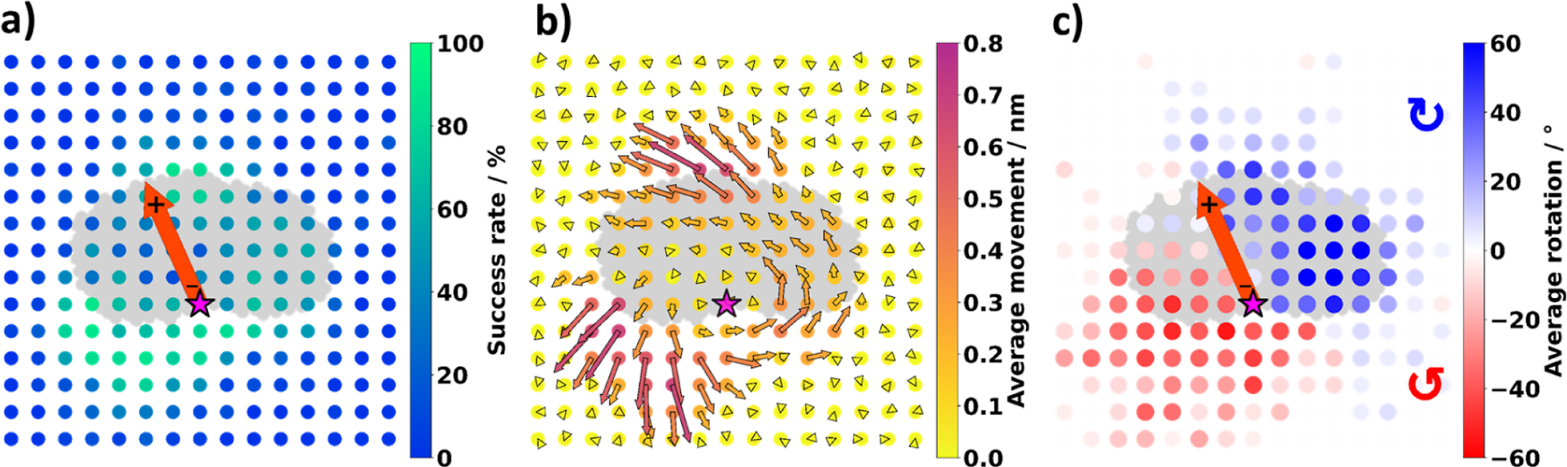
Translational and rotational behavior of the molecule in the presence of an E-field by positioning the STM-tip on the grid. (a) Success rate for inducing a movement of the molecules pivot point (*). (b) Average distance and direction the pivot point of the molecule (*) moves are given by the arrows. Both the color of the arrow and their length represent the average movement distance. (c) Average rotation in clockwise (blue) and counterclockwise (red) direction. All averages were taken over all actions, including unsuccessful attempts.

In addition to the single-molecule manipulation, which requires us to keep track of the molecule with sub-nanometer precision at every manipulation step, our approach also allows us to autonomously generate data leading to physical insight and an understanding of the interaction between the molecule and the electric field induced by the STM tip. The molecule’s position and orientation are tracked and allow us to reveal the molecular behavior. For easy explanation of the molecule’s behavior, the movement directions of the molecule are named by starting at the 12 o’clock position and continue in clockwise direction leading us to the 3, 6, and 9 o’clock position. As summarized in Figure 5, the rotational and translational behavior of the molecule is dependent on the position of the dipole axis. Figure 5a shows the likelihood of inducing a translation, and we find that proximity of the tip to NO_2_ and N(CH_3_)_2_ (approximately 6 and 12 o’clock) has a high chance of success, in agreement with what was observed previously.^39^ Note that this differs from the definition of a successful movement used in Figure 3, where the requirement was that the molecule moves toward the goal (the action incurred a positive reward). We additionally note a high chance of inducing translation further away from the contour of the molecule at 7–8 o’clock positions (Figure 5b)—positions which were not sampled previously. The physical insight provided by Figure 5b,c is that the direction of motion of the molecule is dictated entirely by the position of the tip with respect to the dipole axis, correspondingly implying that the main mechanism is electrostatic in nature and not due to other processes such as inelastic tunneling. A discussion of the dynamics of each induced motion has been given previously where data were acquired manually.^45^

Throughout this study, the agent performed a total of 12,379 actions, providing us with rather accurate statistics. We note in passing that, due to the way we set up the algorithm, not each point of the action space was sampled equally often. Points that are likely to induce a positive reward are visited more frequently (as the agent chooses the actions that presently seem to be the best during the validation runs), while points that induce negative rewards are only sampled during training when exploring the action space with a probability of 70%. Still, every point is visited at least 13 times during the experiment. More details on how often which point was visited are given in Figure S4 of the Supporting Information.

Moreover, we find that near the nitro-group, the region where successful movements can be triggered is more extended, whereas near the dimethylamino group, it is relatively narrow. We speculate that this is a physical effect that is directly related to the geometry of the molecule on the surface. Because the nitro group is closer to the surface than the N(CH_3_)_2_ group,^39^ the nitro group experiences a larger field gradient from the tip even when the tip is at a larger horizontal distance.

Another interesting behavior is revealed by analyzing the directionality of the movement. Figure 5b shows in which direction, and how far, the molecule moves when the tip is located at a certain position. Generally, we find that locations that are likely to induce a successful movement are also likely to move the molecule over a large distance. Some actions are intuitive: for instance, when one wants the molecule to move toward the 6 or 7 o’clock position, efficient actions exist by placing the STM tip at these 6 or 7 o’clock positions of the molecule, respectively. Conversely, other directions of movement are highly unintuitive: for instance, moving the molecule toward the 1 or 2 o’clock position can only be achieved by applying a voltage pulse at the 5 or 6 o’clock position of the molecule. Even then, the action is highly inefficient and only induces small movements.

In principle, it seems possible that a different tip shape influences the interaction. Since in this work, all manipulations were done with the same tip, we cannot rule out this possibility. However, from the analysis of the molecular behavior (Figure 5) where 12,379 manipulations are performed, it can be seen that there is no discernible difference moving the molecule clockwise or counterclockwise along the squared-training trajectory (i.e., the molecule inevitably positioned anywhere around the tip), and we can conclude that the molecular interactions we encountered are independent of the tip asymmetry.

These results are in agreement with those some of us obtained in previous experiments, where the translation and rotation of the molecule are measured at 8 points around the perimeter of the molecule.^39,40,45^ In the present work, the observation space is more extended and higher resolved as the whole action space (i.e., 225 positions) is measured larger and better resolved. Another major advantage is that we could obtain these results automatedly within a couple of days building upon very little prior information (we took the bias voltage and the tip height optimized in previous experiments, and we restricted ourselves to defect-free, flat surfaces). In comparison, a similar study purely reliant on human operators often requires months of laboratory work.

In contrast to the induced translations, the rotations that the actions incur are nicely symmetric around the dipole, as shown in Figure 5c. As could be expected from a dipole interacting with a point charge (representing the STM tip), the rotation occurs clockwise when the tip is located to the right of the dipole and counterclockwise when it is located to the left.^45^ When the average rotation is larger, the closer the tip is to the negative side of the dipole.

There is a statistical chance that during a run, the molecule rotates into an unfavorable orientation where the goal is in a direction for which the molecule is hard to move toward. When, during a validation run, the molecule coincidentally encounters such a situation, no good actions exist at all and it may take a while to get out of this unfavorable rotation. This explains the large differences in required manipulations observed during different runs in the same validation set. In hindsight, it becomes clear that the reason for this (unwanted) behavior intrinsically lies in the way we designed our reward function, which is unaware of the rotation. Obvious solutions to this would be to either directly encode the rotation in the reward function, or to use bootstrapping (which is a technique where instead of giving the reward immediately for the current action, rewards are summed over subsequent actions, thus favoring an initial action that end with favorable rotation but without translation), or alternatingly perform a rotation of the molecule (with a voltage of 1.3 V) and a translation (with a voltage of 1.7 V, see ref (34)) afterward. In general, with the learnings from the previous experiments, it would be possible to design an improved reward function, for example, by lowering the penalty for small movements and increasing the reward for larger movements, possibly in addition to using two separate reward functions for rotations and translations. A systematic approach would be to use inverse reinforcement learning to determine the reward function automatically.^46^ However, the design of an ideal reward function to optimize the performance of the agent would be a separate study in itself and is beyond the scope of this work.

## Conclusions

In this work, we have set up an autonomous reinforcement learning algorithm to efficiently control a molecule across Ag(111) utilizing a tip-induced electric field. The manipulation occurs by placing the tip in an automatically selected position relative to the molecule and applying a voltage pulse at a fixed tip height above the surface. The training progresses extremely fast; at 700 training iterations, the manipulations become efficient, which move the molecule with a probability of 72% for an average distance of 0.58 nm toward the goal. In the final validation run (after 2250 training iterations), our agent manipulates the molecule with 82% probability toward the individual goals and reaches an average translation distance of 0.77 nm per manipulation. Our tests show that the learned behavior is not restricted to the training trajectory (i.e., four goal positions forming a square), but that arbitrary trajectories on the surface can be traversed.

We expect that this opens up the possibility to assemble molecules in multiple constructs, when combining our algorithm with pathfinding algorithms and image recognition software that finds the optimal pathway while avoiding surface defects, or adsorbates, as well as other possible obstacles on which the molecule would get stuck.^36,47,48^

The most critical part of the algorithm is the design of the reward function. Due to the way we designed the function in this work, it penalizes unsuccessful actions more heavily than rewarding successful actions; the agent initially trades off actions that are reliable for actions of smaller moving distance and vice versa. However, after about 2000 learned iterations, the agent learns actions that are more reliable while also moving the molecule across a larger distance. A remaining challenge is that in some situations, no single action exists that performs well. With the data at hand, the clear solution to this is to implement bootstrapping, which allows to evaluate the outcome of several combined actions. Although bootstrapping increases the learning effort, it enables the agent to learn more complex manipulations. Another possible solution would offer an action space expansion to negative bias voltages but increases the learning effort, as every voltage parameter added to the repertoire of the agent increases the action space by 225 entries.

A significant advantage of our approach is that this allows a post-hoc analysis of the decision process of the algorithm. Furthermore, it yields physical insight into what molecular behavior is induced when applying an electric field at points in its close vicinity. This leads to a deeper understanding of how polar molecules move through the potential energy landscape at interfaces. This study can build the foundation in adopting artificial intelligence to learn complex molecular behaviors. The augmentation of single-molecular manipulations with path planning^47,48^ and image recognition algorithms will generate an algorithm capable of autonomous molecular assembly,^49−51^ building the basis for future, bottom-up constructions of artificial and functional structures relevant for nanotechnology applications.

## Supporting Information Available

Molecule extraction; molecule topography determination; example of rotational behavior; sampled action space distribution; exploration *via* Gaussian process regression; interface explanation between the machine learning algorithm, and the STM software (PDF)
