# Supplementary material for: Autonomous Single-Molecule Manipulation Based on Reinforcement Learning
Source: J Phys Chem A. 2023 Feb 7;127(8):2041–50. doi: 10.1021/acs.jpca.2c08696 (PMC9986865; doi:10.1021/acs.jpca.2c08696)
Supplement: Supplementary file 1 — jp2c08696_si_001.pdf [file jp2c08696_si_001.pdf]

Autonomous Single-Molecule Manipulation Based on Reinforcement Learning

Bernhard Ramsauer[1], Grant J. Simpson[2], Johannes J. Cartus[1], Andreas Jeindl[1], Victor García-López[3], James M. Tour[4], Leonhard Grill[2], Oliver T. Hofmann[1*]

1 Institute of Solid State Physics, NAWI Graz, Graz University of Technology, Graz, 8010, Austria

2 Department of Physical Chemistry, Institute of Chemistry, NAWI Graz, University Graz, Graz, 8010, Austria

3 Departments of Chemistry, Louisiana State University, Baton Rouge, LA, 70803, USA

4 Departments of Chemistry and Materials Science and NanoEngineering, and the Smalley-Curl Institute and NanoCarbon Center, Rice University, Houston, TX, 77005, USA

Contents

Extracting individual molecules from an island on a Ag(111) surface 2

Analyzing the molecular topography to determine the agent's state 2

Favorable or unfavorable rotations for either large or small movements........ 4

Statistical distribution of the action space sampled by the agent 5

Gaussian Process Regression (GPR) used for exploration........ 6

The interface between the machine learning algorithm and the STM software 6

Extracting individual molecules from an island on a Ag(111) surface

In order to move single DDNB molecules without physical contact, molecules were extracted from islands formed of mostly pure molecules along the step edges of the Ag(111) surface. The extraction is done by performing lateral manipulation with parameters of 0.01 V and 300 pA that move the STM tip very close to the surface. After a single-molecule is extracted, the algorithm can start the learning procedure by maneuvering the molecule over the surface.

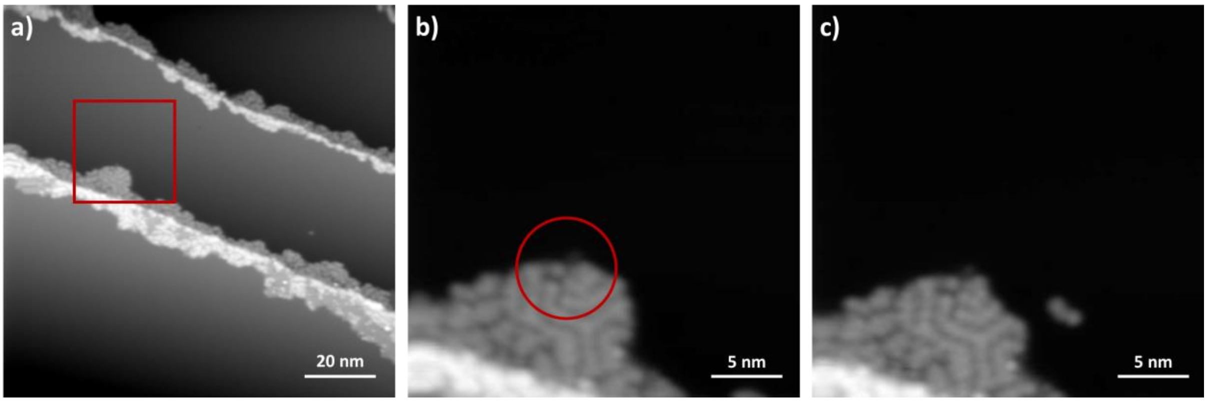

Figure S1: Molecule extraction from an island. (a) Overview image of molecules forming islands along the step edges of the Ag(111) surface. The molecules are extracted from the island in the red square. **(b)** Island of mostly pure molecules with an impurity shown at the top of the island (red circle). **(c)** The molecule is extracted via lateral manipulations (0.01 V and 300 pA) performed on the boarder of the island. STM images: (1.00 V, 11 pA)

Analyzing the molecular topography to determine the agent's state

The molecular information required for the agent to control the molecules, is a) the molecule's position i.e., the pivot point of the molecule (pink star), and b) the dipole orientation (orange arrow). Together with the goal position relative to the molecule, this determines the agent's *state*. This information is obtained autonomously by our Python code by analyzing the molecule's topography, which is measured from an STM-image of size (6.8 x 6.8) nm at a resolution of (64 x 64) pixels, as shown in Figure S2a. The position and dipole orientation of the molecules are determined with our molecule detection algorithm that creates a truncated topography, where the background is subtracted, and the molecule is accentuated (Figure S2d). This allows to determine the molecule's center of mass and the contour to finally obtain the position and orientation as follows:

The <u>**position of the molecule**</u>, that is, the pivot point of the molecule (pink star) is determined by the contour point with the smallest distance from the molecule's center of mass.

The **dipole orientation of the molecule** is determined indirectly from the measured topography. Since the molecule is rigid, it can be derived from the orientation of the molecule's mass axis, adding an offset of 114°. We note that the intrinsic dipole moment causes the STM image to be slightly bumped at the positively charged -$(CH_3)_2$ groups, which is hardly visible in the STM image but revealed in the contour of the molecule as shown in Figure S2 c). This small bump allows us to determine which enantiomer of the molecule is present in the image.

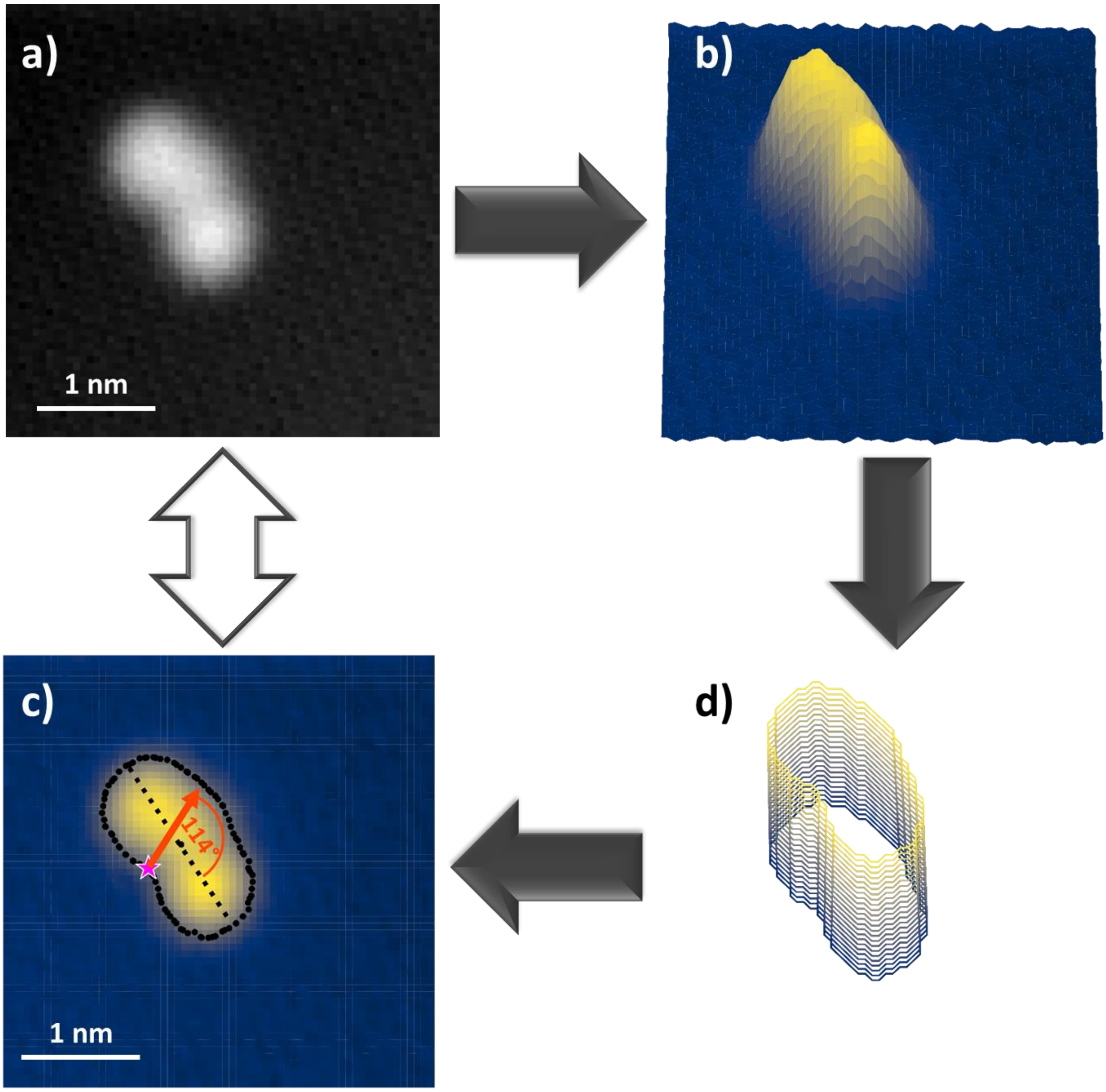

Figure S2: Autonomous topography analysis. (a) Shows the STM-image taken at every manipulation steps. **(b)** Shows the corresponding topography of the STM-image. **(d)** The topography of the molecule is highlighted, and the background removed, leading to a truncated topography. **(c)** The position (pink star), the dipole orientation (orange arrow) and the contour of the molecule are determined from the truncated topography. STM-image: (1.00 V, 11 pA).

Favorable or unfavorable rotations for either large or small movements

The constant spread in the translation distance can be explained by taking a closer look into the movement and especially the rotation of the molecule. Figure S3 shows four timesteps (t=3 to t=6) of successive successful manipulations. The molecule's location before and after applying a voltage pulse (i.e., the tip position indicated by the orange square), is shown by the light red and red colored contour, respectively. The distance the molecules moves (i.e., the movement of the pivot point) is given by the colored line.

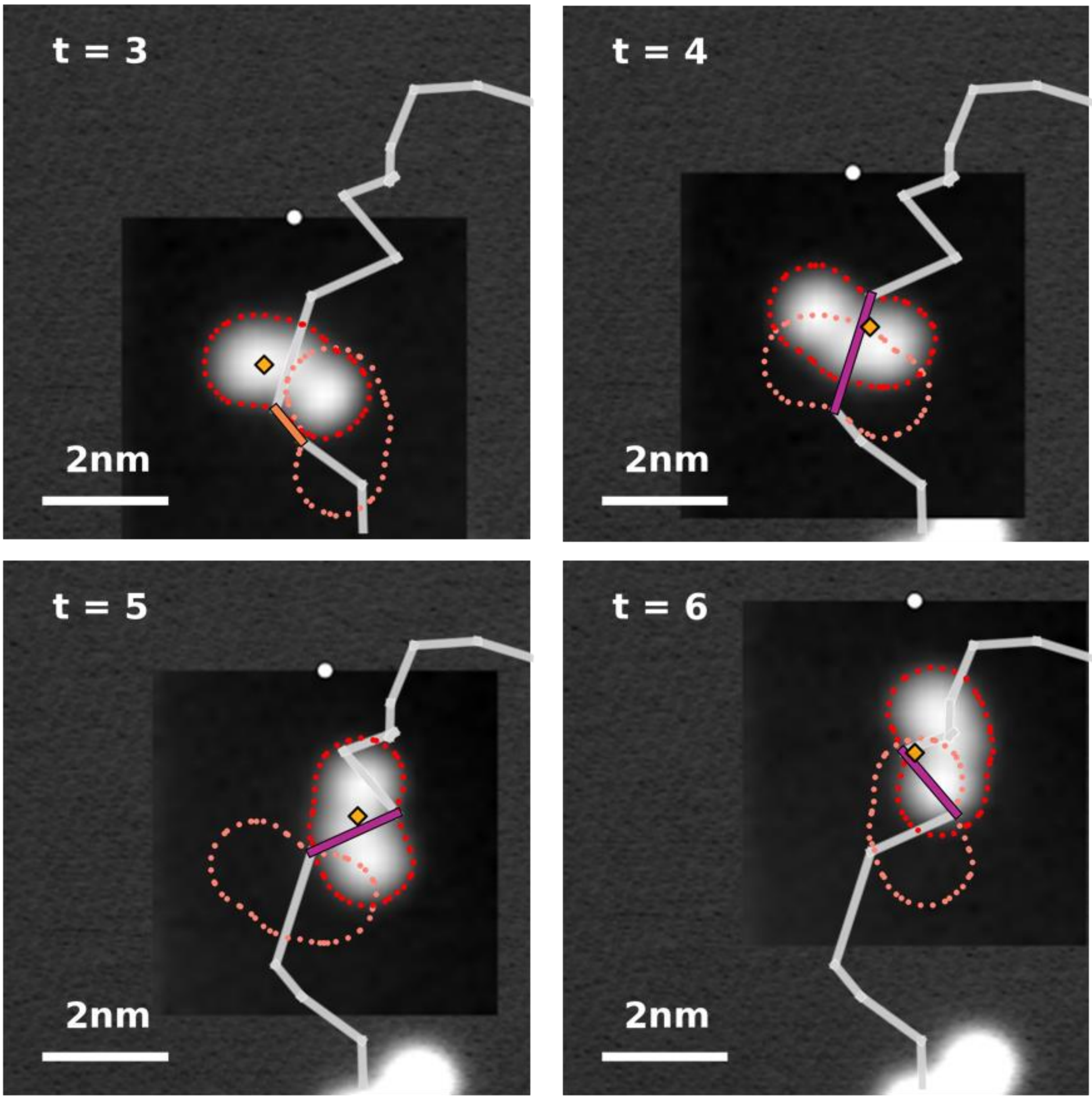

Figure S3: Distance of large movement is mostly possible when the molecule is rotating favorably. The grey line represents a neckline of the trajectory for four timesteps. The light-grey contour of the molecule shows the orientation before the voltage pulse was applied. In timestep **(t=3)** the molecule rotates for +60 °, which is a rotation into the previous position, and is leading to a smaller movement of 0.70 nm compared to the following movements. In the next manipulation step **(t=4)** the molecule favorably rotates by 180 ° and moves for about 2.04 nm. At timestep **(t=5)** the molecule rotates by -60 ° but away from the previous position and moves for 1.60 nm. At timestep **(t=6)** the molecule again rotates by 180 ° and translates upwards for a total movement of 1.40 nm. The yellow square shows the tip position where the voltage pulse is applied. STM images: (1.00 V, 11 pA)

At timestep **t=3**, the molecule is rotating inwards with respect to the pivot point which leads to a smaller distance the molecule moves. In the next timestep **t=4**, the molecule rotates by 180 ° which contributes favorably to the distance the molecule moves. At timestep **t=5**, the molecule is favorably rotating and translating away from the previous position of the pivot point leading to a large distance moved. At the last timestep shown **t=6**, the molecule again rotates favorably by 180 ° and the distance moved is quite large. This shows that the distance the molecule moves depends on the rotation involved.

Statistical distribution of the action space sampled by the agent

The agent manipulates the molecule based on the action space (i.e., a regular grid relative to the molecules center). The number of times the agent sampled the individual actions are shown in Figure S4. The success rate and the number of times an action is performed are directly linked with each other because the higher the success rate of an individual action is the higher the accumulated reward and the more often this action is selected by the agent.

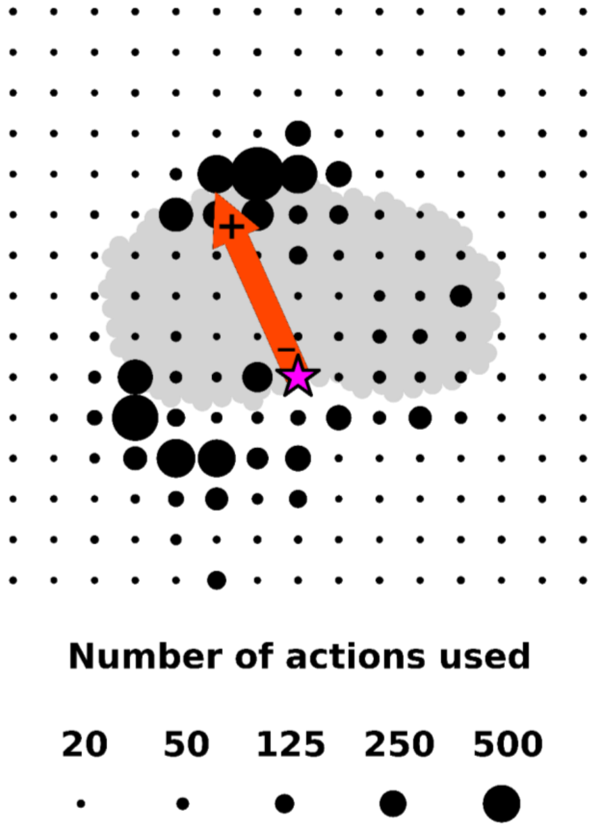

Figure S4: Statistical distribution for the individual points of the action space. The dot-size of an action is equivalent to the number of times the agent performed this action. The total number of performed actions are 12.379 and the lowest number an action is performed is 13. The molecule is shown in grey based on the measured STM topography. The size of the squared action space is 4.2 x 4.2 nm.

Gaussian Process Regression (GPR) used for exploration

The agent's action space is modeled via GPR using squared exponential kernels (eq. 1). When exploring the environment, the agent uses the action of highest GPR uncertainty. This allows the agent to use the most informative action.

$$k_{SE}(\mathrm{x},\mathrm{x}') = \sigma^2 \exp\left(-\frac{(x-x')^2}{2l^2}\right) + \delta_{ij}\sigma_{noise}^2 \qquad (1)$$

The signal variance $\sigma > 0$ is a scaling factor that determines the variation from the mean. The length scale $l > 0$ determines how smooth the training data can be fitted but also how far and reliable we can extrapolate from the training data. The noise covariance $\sigma_{noise} \geq 0$ is factored by the Kronecker delta δ_{ij} and is set to 0.1. The two kernel hyperparameters for the signal variance σ and length scale l are set to 1 and 0.0952 respectively. Therefore, the length scale of a kernel is about two times the distance between two neighboring action positions, and the signal variance was simply set to the maximum possible reward. Since we do not use the predicted GPR values for selecting an action, but only the GPR uncertainty, this scaling factor does not influence our agent's decision.

The interface between the machine learning algorithm and the STM software

The STMAFM software is developed by CreaTec utilizing a key, value-based interface command structure that allows to access specific parameters. The interface can be accessed by OLE/COM interface which stands for Object Linking and Embedding and Component Object Model. The OLE interface is an object system protocol that allows applications that are OLE capable to communicate with each other. The COM interface is developed for the Windows operating system for inter-process communication (IPC).
